# Metagenomic and phytochemical analyses of kefir water and its subchronic toxicity study in BALB/c mice

**DOI:** 10.1186/s12906-021-03358-3

**Published:** 2021-07-01

**Authors:** Muganti Rajah Kumar, Swee Keong Yeap, Nurul Elyani Mohamad, Janna Ong Abdullah, Mas Jaffri Masarudin, Melati Khalid, Adam Thean Chor Leow, Noorjahan Banu Alitheen

**Affiliations:** 1 Department of Cell and Molecular Biology, Faculty of Biotechnology and Biomolecular Sciences, Universiti Putra Malaysia, UPM, 43400 Serdang, Selangor Darul Ehsan Malaysia; 2 China-ASEAN College of Marine Sciences, Xiamen University Malaysia, 43900 Sepang, Malaysia; 3 Biotechnology Research Institute, Universiti Malaysia Sabah, 88400 Kota Kinabalu, Sabah Malaysia; 4 UPM-MAKNA Cancer Research Laboratory, Institute of Bioscience, Universiti Putra Malaysia, 43400 Serdang, Selangor Darul Ehsan Malaysia; 5 Department of Biomedical Sciences, Universiti Putra Malaysia, UPM, 43400 Serdang, Selangor Darul Ehsan Malaysia

**Keywords:** Kefir water, 16S rRNA, UHPLC, Toxicity, Antioxidants

## Abstract

**Background:**

In recent years, researchers are interested in the discovery of active compounds from traditional remedies and natural sources, as they reveal higher therapeutic efficacies and improved toxicological profiles. Among the various traditional treatments that have been widely studied and explored for their potential therapeutic benefits, kefir, a fermented beverage, demonstrates a broad spectrum of pharmacological properties, including antioxidant, anti-inflammation, and healing activities. These health-promoting properties of kefir vary among the kefir cultures found at the different part of the world as different media and culture conditions are used for kefir maintenance and fermentation.

**Methods:**

This study investigated the microbial composition and readily found bioactive compounds in water kefir fermented in Malaysia using 16S rRNA microbiome and UHPLC sequencing approaches. The toxicity effects of the kefir water administration in BALB/c mice were analysed based on the mice survival, body weight index, biochemistry profile, and histopathological changes. The antioxidant activities were evaluated using SOD, FRAP, and NO assays.

**Results:**

The 16S rRNA amplicon sequencing revealed the most abundant species found in the water kefir was *Lactobacillus hilgardii* followed by *Lactobacillus harbinensis*, *Acetobacter lovaniensis*, *Lactobacillus satsumensis*, *Acetobacter tropicalis*, *Lactobacillus zeae*, and *Oenococcus oeni*. The UHPLC screening showed flavonoid and phenolic acid derivatives as the most important bioactive compounds present in kefir water which has been responsible for its antioxidant activities. Subchronic toxicity study showed no toxicological signs, behavioural changes, or adverse effects by administrating 10 mL/kg/day and 2.5 mL/kg/day kefir water to the mice. Antioxidants assays demonstrated enhanced SOD and FRAP activities and reduced NO level, especially in the brain and kidney samples.

**Conclusions:**

This study will help to intensify the knowledge on the water kefir microbial composition, available phytochemicals and its toxicological and antioxidant effects on BALB/c mice since there are very limited studies on the water kefir grain fermented in Malaysia.

## Introduction

Kefir is a self-carbonated refreshing fermented beverage that has been consumed over 100 s of years for its various health benefits. It is originated by communities in the Balkans in Eastern Europe and the Caucasian mountains in Central Asia [1, 2]. The beverage has a unique flavour due to its viscous texture with an acidic and tart taste, low levels of alcohol, and other fermentation flavour products. The feature that distinguishes kefir from other fermented dairy products is the use of kefir grains during fermentation and the presence of various kinds of microbial flora, predominantly lactic acid bacteria, acetic acid bacteria, yeasts, and fungi [3–5]. The most commonly found bacterial genera in kefir grains are *Lactobacillus, Bifidobacterium, Oenococcus, Lactococcus, Streptococcus,* and *Leuconostoc* [1, 6–8]. This makes kefir a great probiotic beverage. Besides, kefir promotes a wide range of health benefits including anti-bacterial, anti-fungal, anti-tumour, anti-inflammatory, healing, anti-carcinogenic, immune system stimulatory, hypocholesterolemic, and contains high antioxidant properties [1, 9–18]. Nevertheless, these numerous health benefits of kefir vary among each kefir cultures found all over the world. This is because of the usage of different media and culture conditions used during kefir maintenance and fermentation. Besides, in recent times, people in Malaysia have started to acknowledge and consume kefir as a daily probiotic supplement. However, there are very limited records on microbiota composition of kefir grain and the available metabolites present in kefir water fermented locally, and its toxicity profile and antioxidant activity by oral administration in-vivo.

Next-generation sequencing metagenomics (NGSm) is a high throughput sequencing method which comprised of exclusively selected gene markers such as 16S rRNA that has been most widely applied to study the taxonomic composition of microbial populations. In recent times, this technique has been used widely in spontaneous or controlled food fermentation processes such as kefir, kimchi, wine, and fermented dairy products to identify the unique microbial community dynamics [19–23]. The general process to perform NGSm analysis on food products involves (1) DNA isolation, (2) specific marker gene(s) PCR amplification, (3) preparation of sequencing libraries by introducing barcodes and sequencing platform adapters, (4) generating millions of reads per sample using NGS, and (5) processing sequencing reads and bioinformatics tool analysis [24]. Among the most reliable and powerful bioinformatics analytical software for successful NGSm study are Mothur, QIIME, SILVA, MEGAN, and Amplicon Noise [25]. NGSm helps in improving and optimising food production processes by studying the relationship between bacterial communities’ compositions and its quality of the end products, which includes the flavour, aroma, and nutritional properties. Studies by Wang et al. have identified aromatic compounds producing core bacteria during vinegar fermentation using the microbial communities’ composition [26, 27].

Phytochemicals are non-nutrient bioactive compounds produced mainly by plants and thus found in plant-based foods, including grains, vegetables, and fruits. The phytochemicals can be categorized into various groups such as polyphenols, alkaloids, carotenoids, organosulfur compounds, and nitrogen-containing compounds [28, 29]. The most studied compound is polyphenols and it can be further classified into flavonoids (including flavones, flavonols, flavanones, isoflavones, catechins, and anthocyanidins), phenolic acids, tannins, stilbenes, and coumarins [28, 29]. These compounds are the main source for single bioactive ingredients in modern and nutraceuticals medicines. They have great antioxidant properties and exhibit potent protective effects against various infectious and chronic diseases [28–30]. The presence of phytochemicals in a sample can be detected by high-performance liquid chromatography (HPLC), gas chromatography (GC), or capillary electrophoresis (CE). Following sample pre-separation, the compounds are separated from one another and identified by different means using mass spectrometry (MS) or nuclear magnetic resonance (NMR) [31]. This study investigated kefir water phytochemicals that are present using ultra-high-performance liquid chromatography (UHPLC). UHPLC is a rising chromatographic separation technique that has been successfully employed for high chromatographic resolution, speed, and with the required sensitivity. This system takes full advantage of chromatographic principles to run separation using packing materials with smaller particular size lesser than 2.5 μM and contains high linear velocity mobile phases that reduce the length of the column as well as the solvent consumption and saves time [32]. TWINS-QTOFMS analysis helps in the compound structure elucidation and identification of compound fragmentation patterns [33–35]. This coupling of QTOFMS with liquid chromatography technique provides both comprehensive and rapid screening solutions that become prominent in the clinical laboratory setting.

The present study report for the first time the evaluation of dynamic microbial community and their available metabolites in kefir water fermented in Malaysia. The study further investigates the toxicological profiles and the antioxidant activities of the kefir water on BALB/c mice. Even though kefir water has been acknowledged and consumed traditionally in Malaysia, reports on its safety of consumption and its beneficial effects to the consumers are very limited. Thus, in this study, we attempted to investigate the oral subchronic toxicity of this kefir water in BALB/c mice, which would be crucial to justify the safety of consuming this kefir water. Furthermore, the data from this study will also help to commercialise kefir water in Malaysia.

## Materials and methods

### Preparation of kefir water

Kefir grain was obtained from a local store known as Kefir and Kombucha in Kuala Lumpur, Malaysia (https://www.kefirandkombucha.com). Eighteen grams of kefir grain was added into a glass vessel and mixed with 50 g of organic raw sugar and 500 mL of mineral water. The kefir was left to ferment for 24 h at room temperature. Then, the kefir grain was filtered out from the fermented medium using 110 mm filter paper (Whatman, England). The kefir water was then stored at -20 °C prior to usage. Kefir water was made once in every 2 days to ensure its freshness.

Metagenomics analysis of kefir grain using IonS5™XL NGS.

### Extraction of genome DNA

Twenty milligrams of kefir grain were ground finely and added 1 mL of pre-heated (60 °C) CTAB lysis buffer. The CTAB buffer was made by mixing 20 mM sodium EDTA with 100 mM Tris-HCL (pH 8) and then, 1.4 M NaCl and 20% (w/v) CTAB (cetyltrimetthylammonium bromide) were added (0.2% of β-mercaptoethanol was added right before use). The kefir sample was then incubated in a water bath at 60 °C for 1 h. The sample was mixed well in every 15 min. Chloroform:isoamyl (C:I) (500 mL) was added, mixed and then centrifuged at 13,226 x g for 10 min. Then, the supernatant was collected. The extraction step was repeated until a clear inter-phase was obtained. Five microlitres of RNAse (10 mg/mL) was added to the supernatant and incubated at 37 °C for 1 h. The extraction step was carried out once at this step. An equal volume of isopropanol was added to the sample, mixed and incubated on ice for 15 min. Then, the sample was centrifuged at 13,226 x g for 10 min. The supernatant was discarded, and the DNA pellet was collected. The pellet was washed with 70 °C ethanol and dried with air prior to dissolving in 30 μL TE buffer.

### Amplicon generation

The 16S rRNA genes of distinct regions (16SV4/16SV3/16SV3-V4/16SV4-V5) were amplified using specific primers such as 16S V4: 515F-806R with the barcode. All the PCR reactions were carried out with Phusion® High-Fidelity PCR Master Mix (New England Biolabs).

### PCR products quantification and qualification

The same volume of 1X loading buffer containing SYB green was mixed with PCR products and operated electrophoresis on 2% agarose gel for detection. Samples with a bright main strip between 400 and 450 bp were chosen for further experiments.

### PCR products mixing and purification

The libraries were generated with Ion Plus Fragment Library Kit 48 rxns for Thermofisher and quantified via Qubit and Q-PCR, was sequenced by IonS5TMXL (Thermofisher).

### Sequencing data processing

Single-end reads were assigned to samples based on their unique barcode and truncated by cutting off the barcode and primer sequence. Quality filtering on the raw reads were performed under specific filtering conditions to obtain the high-quality clean reads according to the Qiime (V1.7.0, http://qiime.org/scripts/split_libraries_fastq.html) quality-controlled process. The reads were compared with the reference database (Gold database, http://drive5.com/uchime/uchime_download.html) using UCHIME algorithm (UCHIME Algorithm, http://www.drive5.com/usearch/manual/uchime_algo.html) to detect chimera sequences. Then, the chimera sequences were removed. The Effective Reads were finally obtained.

### OTU cluster and species annotation

Sequences analysis were performed by Uparse software (Uparse v7.0.1001 http://drive5.com/uparse/) using all the effective reads. Sequences with ≥97% similarity were assigned to the same OTUs. Representative sequence for each OTU was screened for further annotation. For each representative sequence, Mothur software was performed against the SSUrRNA database of SILVA Database (http://www.arb-silva.de/) for species annotation at each taxonomic rank (Threshold:0.8 ~ 1) (kingdom, phylum, class, order, family, genus, species). To get the phylogenetic relationship of all OTUs representative sequences, the MUSCLE (Version 3.8.31, http://www.drive5.com/muscle/) can compare multiple sequences rapidly. OTUs abundance information was normalised using a standard of sequence number corresponding to the sample with the least sequences.

### Ultra high-performance liquid chromatography (UHPLC)

UHPLC was performed on ACQUITY UPLC I-Class system from Waters, consisting of a binary pump, a vacuum degasser, an autosampler and a column oven. Phenolic compounds were chromatographically separated using a column ACQUITY UPLC HSS T3 (100 mm × 2.1 mm × 1.8 μm), also from Waters, maintained at 40 °C. A linear binary gradient of water (0.1% formic acid) and acetonitrile (mobile phase B) was used as mobile phase A and B, respectively. The mobile phase composition was changed during the run as follows: 0 min, 1% B; 0.5 min, 1% B; 16.00 min, 35% B; 18.00 min, 100% B; 20.00 min, 1% B. The flow rate was set to 0.6 mL/min, and the injection volume was 1 μL.

The fragmentation is based on the first mass quadruple in the mass spectrometer where the parent mass or compound are decided i.e. through its mass over charge ratio (m/z), filter by the quadruple at the first stage i.e. Q1, and then directed into the collision cell of Q2 where fragmentation happened. After that, it passed through a tube called the time of flight where the mass is then directed ‘slower’ to the detector to get the electronic signal. The slower or longer path for the ions to travel to the detector will give the system more time to ‘look at the mass’ hence obtaining a better mass accuracy.

### TWINS-QTOF-MS analysis

The UHPLC system was coupled to a Vion IMS QTOF hybrid mass spectrometer from Waters, equipped with a Lock Spray ion source. The ion source was operated in negative electrospray ionisation (ESI) mode under the following specific conditions: capillary voltage, 1.50 kV; reference capillary voltage, 3.00 kV; source temperature, 120 °C; desolvation gas temperature, 550 °C; desolvation gas flow, 800 L/h, and cone gas flow, 50 L/h. Nitrogen (> 99.5%) was employed as desolvation and cone gas. Data were acquired in high-definition MSE (HDMSE) mode in the range m/z 50–1500 at 0.1 s/scan. Thus, two independent scans with different collision energies (CE) were alternatively acquired during the run: a low-energy (LE) scan at a fixed CE of 4 eV, and a high- energy (HE) scan where the CE was ramped from 10 to 40 eV. Argon (99.999%) was used as collision-induced-dissociation (CID) gas.

### Experimental animals

A total of 12 male BALB/c mice were used in this study. The in vivo subchronic study was performed according to the OECD guidelines with the approval of the Universiti Putra Malaysia Animal Care and Use Committee (UPM/IACUC/AUP-R045/2018). The study was carried out in compliance with the ARRIVE guidelines. Animals were purchased from the Animal House of the Faculty of Veterinary Sciences, Universiti Putra Malaysia. Male BALB/c mice of 18–20 g weight and age of 5–6 weeks were used in this study. The mice were placed in plastic cages at 22 ± 1 °C with 12 h of dark/light cycle with relative humidity approximately 60%. Upon purchase, the mice were acclimatised for 7 days and received standard pellet diet and distilled water ad libitum throughout the experiment.

### Subchronic toxicity study

A total of 12 male BALB/c mice of 5–6 weeks’ age were divided into three groups; control group (*n* = 4), kefir 10 mL/kg BW treated group (*n* = 4), and kefir 2.5 mL/kg BW treated group (*n* = 4). Kefir samples were administered orally through oral gavage daily for 28 days. The high and low dosages of kefir water were prepared fresh every day prior to the treatment time. The low dosage kefir (2.5 mL/kg BW) was diluted with distilled water prior to the treatment. The mice were monitored daily upon treatment until sacrificed. Blood serum and crucial organs such as the liver, kidney, spleen, and brain were collected for the biochemical analysis, histopathological analysis, and antioxidant assays.

### Clinical observations and body weight change

The mice were observed every day upon treatment for any changes in the general physical conditions such as appearance, behaviour, fur condition, and mortality. The body weight was measured every 7 days using a tabletop electronic balance.

### Biochemical analysis

At the end of the treatment duration, the mice were fasted for 3 h and then sacrificed by anaesthesia with ketamine-xylazine (100 mg/kg:10 mg/kg) through intraperitoneal injection. Then, the blood was drawn from the mice heart using heparin-coated syringes and dispensed into microtainer tubes (Becton Dickinson, USA). The collected blood samples from each mouse were spun at 14000 x g for 15 min at 4 °C. Then, the serum was transferred from the microtainer tubes into 1.5 mL centrifuge tubes and kept in a freezer at -20 °C until use. The serum from each mouse was analysed using the ELISA assay kits (Roche, Germany). The levels of serum aspartate aminotransferase (AST), alanine aminotransferase (ALT), alkaline phosphatase (ALP), gamma glutamyltranspeptidase, albumin and total protein were evaluated for hepatic function. For renal function, the levels of serum creatinine and urea were examined.

### Histopathological analysis

Organs such as the liver, kidney, spleen, and brain were harvested and weighed. Half of each organ was used for the hematoxylin and eosin staining. First, the tissues were subjected to fixation and then the tissues were cut into smaller pieces and put into cassettes. The tissues were then processed using an automated tissue processor (Leica TP 1020; Leica Biosystems Nussloch GmbH, Buffalo Grove, IL, USA) where it carries out the dehydration, clearing, and impregnating process. Then, the tissue samples were embedded in molten paraffin. Microtome was used to trim the blocks at 16 μm thickness and sectioned at a thickness of 0.4 μm. The thin sections of the tissue samples were placed in a water bath and fished onto the glass slides. The glass slides were then stained with hematoxylin and eosin followed by p-xylene-bis-pyridinium bromide mounting. The slides were then observed under a light microscope. Different fields from each slide of each group were examined to evaluate the histological changes.

### Antioxidant activity in liver, kidney, spleen and brain samples

#### Superoxide dismutase (SOD) assay

Firstly, a master mix of SOD containing 0.1 mol/L phosphate buffer, 0.15 mg/mL sodium cyanide in 0.1 mol/L ethylene diamine tetraacetic acid (EDTA), 1.5 mmol/L nitro blue tetrazolium (NBT) and 0.12 mmol/L riboflavin was prepared. Then, the liver, kidney, spleen and brain homogenate samples were prepared in a 96-well plate by a 2-fold serial dilution in 100 μL, individually. 200 μL of the master mix was then added into each well-containing sample homogenates. The absorbance was measured at 560 nm using an ELISA plate reader (Bio-Tek Instrument, USA), and the SOD activity was expressed as unit SOD/mg protein.

#### Ferric reducing ability plasma (FRAP) assay

Briefly, a master solution containing 30 mL of 300 mM acetate buffer (pH 4) to 3 mL of 10 mM TPTZ (2, 4, 6-tripyridyl-s-triazine) solution and 3 mL of 20 mM FeCl_3_.6H_2_O solution in 40 mM HCl was prepared. This master solution was warmed at 37 °C and kept from light prior to usage. 150 μL of the master solution and 80 μL of liver, kidney, spleen and brain homogenates were added to the 96-well plate individually and mixed well. The sample mixtures were then incubated for 10 min at room temperature. The absorbance was measured at 593 nm using an ELISA plate reader (Bio-Tek Instrument, USA). The FRAP activity was analysed based on the standard FeSO_4_ (0–100 μM) calibration curve.

#### Nitric oxide (NO) assay

The nitric oxide level in liver, kidney, spleen and brain samples from kefir water treated and untreated groups were measured based on the user’s guideline in the Griess reagent kit (Sigma, USA). The liver, kidney, spleen and brain were mashed individually using plunger and 70 μm cell strainer in phosphate-buffered saline (PBS). The respective supernatants were collected and mixed with deionised water and the provided Griess reagent. The samples were then incubated for 30 min at room temperature. After incubation, the absorbance was measured using a microplate reader (Bio-Tek Instruments, Winooska, VT, USA) at 548 nm. The nitrite concentration in each sample was analysed based on the standard curve of the standard nitric solution.

#### Statistical analysis

All data are presented as mean ± standard deviation (SD). The data were evaluated by one-way analysis of variance (ANOVA) using a statistical package for the social sciences (SPSS) software (SPSS Inc., Chicago, IL). Statistical significance was considered at *p* < 0.05.

## Results

Microbial diversity of 16S rRNA and the taxonomic tree of bacteria at the genus and species level.

**Fig. 1:**
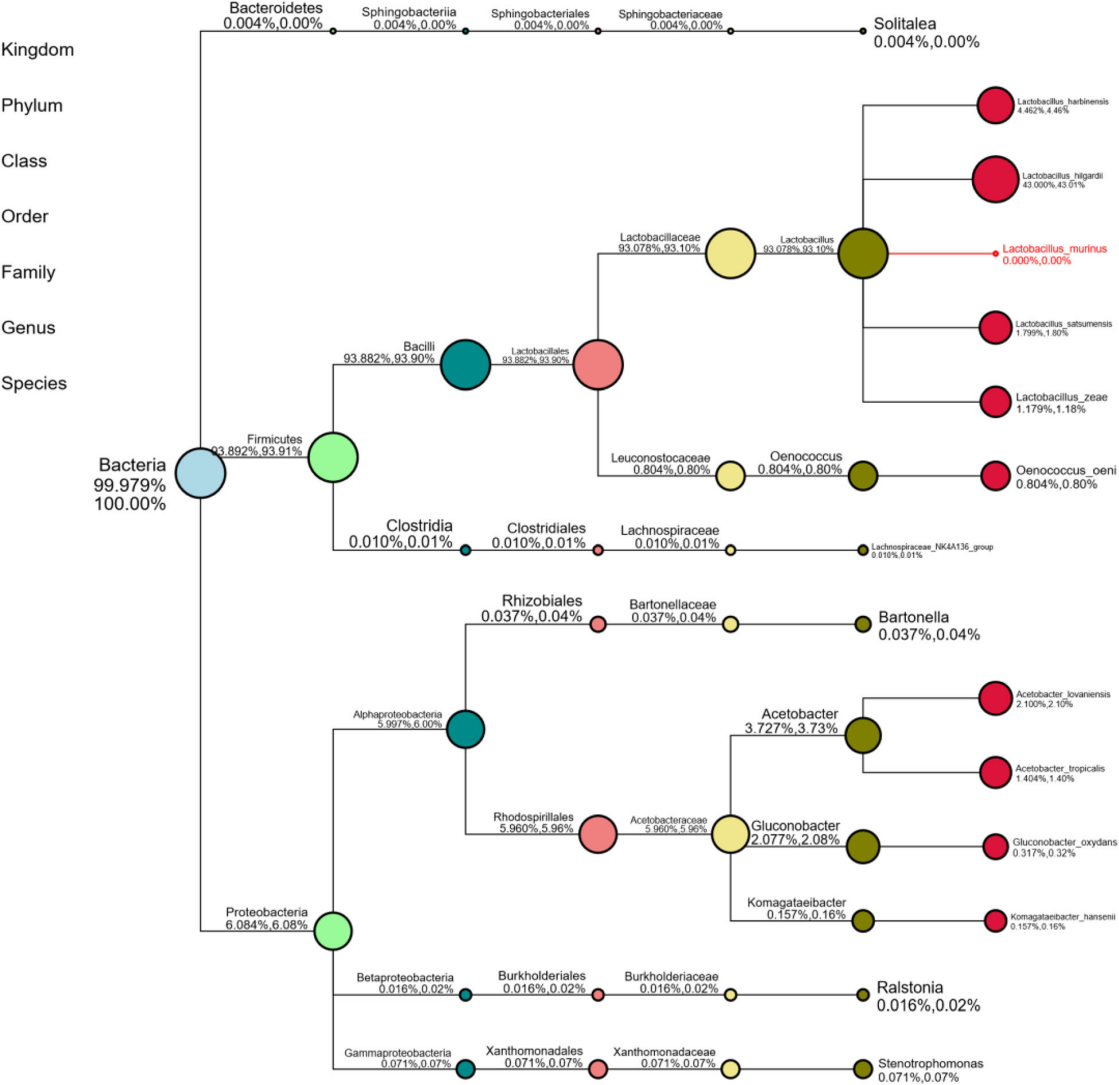
The taxonomical tree obtained for kefir grain at the species level. Notes: Different colours represent different taxonomic ranks. The size of circles stands for the relative abundance of species. The first number below the taxonomic name represents the percentage in the whole taxon, while the second number represents the percentage in the selected taxon

The composition and diversity of the bacterial communities in the kefir grain was determined using the 16S rRNA genes of distinct regions (16SV4/16SV3/16SV3-V4/16SV4-V5). MUSCLE software (Version 3.8.31, http://www.drive5.com/muscle/) was used to obtain the microbial taxonomical classification and display the diversity of kefir grain according to NCBI taxonomy. From the 16S RNA gene fragments, specific species with the top 10 genera in high relative abundance were selected to make the taxonomy tree (Fig. 1) by independently R&D software. The top phylum detected is Firmicutes (93.892%) followed by Proteobacteria (6.084%) and Bacteroidetes (0.004%). Firmicutes phylum is mainly comprised of Bacilli class (93.882%), Lactobacillales order (93.882%), and Lactobacillaceae (93.078%) family. The most abundance genus found in the kefir grain was *Lactobacillus* with 93.078% reads followed by *Acetobacter* (3.727%), *Gluconobacter* (2.077%), *Oenococcus* (0.804%), *Komagataeibacter* (0.157%), *Stenotrophomonas* (0.071%), *Bartonella* (0.037%), *Ralstonia* (0.016%), *Lachnospiraceae_NK4A136_group* (0.010%), and *Solitalea* (0.004%) in trace amounts. The most abundance species found was *Lactobacillus hilgardii* (43%) followed by *Lactobacillus harbinensis* (4.462%), *Acetobacter lovaniensis* (2.1%), *Lactobacillus satsumensis* (1.799%), *Acetobacter tropicalis* (1.404%), *Lactobacillus zeae* (1.179%), *Oenococcus oeni* (0.804%), *Gluconobacter oxydans* (0.317%), and *Komagataeibacter hansenii* (0.157%).

### UHPLC-TWINS-QTOF-MS analysis

The TWINS-QTOF-MS analysis by ChemSpider and PubChem database obtained through the Waters UNIFI Scientific Information System showed that the kefir water used in this study is rich in flavonoids and phenolic contents, as shown in Table 1.

**Table 1:** List of identified compounds

No.	Component name	Formula	Neutral mass (Da)	Observed neutral mass (Da)	Mass error (ppm)	Mass error (mDa)	Observed RT (min)	Isotope Match Intensity RMS Percent
1	Trifloroside	C35H42O20	782.22694	782.228	1.4	1.1	0.67	1400.16
2	Nevadensin-7-O-[α-L-rhamnosyl(1 → 6)]-β-D-glucoside	C30H36O16	652.20034	652.203	4.1	2.7	0.55	657.32
3	Grosvenorine	C33H40O19	740.21638	740.22	4.8	3.6	0.53	609.62
4	6′-O-Benzoyl-4″-hydroxy-3″-methoxypaeoniflorin	C31H34O14	630.19486	630.1947	−0.3	− 0.2	0.65	447.06
5	Sibiricaxanthone A	C24H26O14	538.13226	538.1307	−2.9	−1.6	0.45	254.81
6	(2R,3R)-Taxifolin7-O-α-L-rhamnopyranosyl-(1 → 6)-β-D-glucopyranoside	C27H32O16	612.16903	612.1671	−3.2	−2	1.01	207.66
7	Spinosin	C28H32O15	608.17412	608.1761	3.3	2	0.63	187.22
8	Acacetin-7-O-(6″-O-acetyl)-β-D-glucopyranoside	C24H24O11	488.13186	488.1339	4.2	2.1	0.58	180.44
9	5,7,2′-Trihydroxy-flavanone-4′-O-β-D-glucoside	C21H22O11	450.11621	450.1146	−3.7	−1.7	1.58	177.32
10	5,7,2′-Trihydroxy-flavanone-4′-O-β-D-glucoside	C21H22O11	450.11621	450.1147	−3.5	−1.6	1.32	139.7
11	Shanciol B	C25H26O6	422.17294	422.1711	−4.3	−1.8	6.13	122.37
12	Iridin	C24H26O13	522.13734	522.1347	−5	−2.6	2.61	118.45
13	Helonioside B	C34H40O18	736.22146	736.2196	−2.5	−1.9	2.86	103.45
14	2,3,5,4′-Tetrahydroxystilbene-2,3-O-β-D-glucopyranoside	C26H32O14	568.17921	568.1766	−4.6	−2.6	1.62	95.29
15	2,3,5,4′-Tetrahydroxystilbene-2,3-O-β-D-glucopyranoside	C26H32O14	568.17921	568.1779	−2.3	−1.3	1.75	93.24
16	Erigoster A	C27H26O13	558.13734	558.1352	−3.9	−2.2	5.26	83.91
17	Helonioside B	C34H40O18	736.22146	736.2186	−4	−2.9	2.23	83.6
18	Ginsenoside Ra1	C58H98O26	1210.63463	1210.6386	3.3	4	0.34	82.61
19	Asperuloside tetraacetate	C26H30O15	582.15847	582.156	−4.3	−2.5	1.44	81.49
20	Nortracheloside	C26H32O12	536.18938	536.1896	0.5	0.2	5.66	81.42

### The phenolic acids and flavonoids that were detected in the kefir water are

Trifloroside is widely found in plant particularly in the herb and roots of Gentiana species. Trifloroside is the pre-dominant phytochemical detected in kefir water with isotype match intensity RMS: 1400.16%. The identity of Trifloroside with m/z 781, has the characteristic fragments of m/z 707 [M − H-74] ^−^, 623 [M − H-84] ^−^, and 546 [M − H-77] ^−^.

Nevadensin-7-O-[α-L-rhamnosyl (1 → 6)]-β-D-glucoside is the second dominant phytochemical detected in kefir water with isotype match intensity RMS: 657.32%. The identity of Nevadensin-7-O-[α-L-rhamnosyl (1 → 6)]-β-D-glucoside with m/z 651, has the characteristic fragments of m/z 623 [M − H-28] ^−^, 607 [M − H-16] ^−^, and 546 [M − H-61] ^−^.

Grosvenorine is the main flavonoid compound found in the fruits of the Chinese medicinal plant, *Siraitia grosvenorii* (Swingle) C. Jeffrey. Grosvenorine with m/z of 739 and characteristic fragments of m/z with 675 [M − H-64] ^−^, 567 [M − H-108] ^−^, and 546 [M − H-21] ^−^ was detected.

6′-O-Benzoyl-4″-hydroxy-3″-methoxypaeoniflorin mostly found in *Paeonia delavayi*. It has an m/z of 629 and characteristic fragments of m/z with 578 [M − H-51] ^−^, 577 [M − H-1] ^−^, and 545 [M − H-32] ^−^.

Sibiricaxanthone A belongs to the group of Xanthones, secondary metabolites that most commonly occurring in higher plant families and fungi. Sibiricaxanthone A has an m/z of 537 and characteristic fragments of m/z with 503 [M − H-34] ^−^, 492 [M − H-11] ^−^, and 380 [M − H-112] ^−^.

(2R,3R)-Taxifolin7-O-α-L-rhamnopyranosyl-(1 → 6)-β-D-glucopyranoside commonly found in seeds of medicinal plants such as *Platycodon grandiflorum* A. DE CANDOLLE. It has an m/z of 611 and characteristic fragments of m/z with 583 [M − H-28] ^−^, 580 [M − H-3] ^−^, and 550 [M − H-30] ^−^.

Spinosin is a flavone C-glycoside found in the seeds of *Zizyphus jujuba* var. spinosa. Spinosin has an m/z of 607 and characteristic fragments of m/z with 602 [M − H-5] ^−^, 571 [M − H-31] ^−^, and 525 [M − H-46] ^−^.

Acacetin-7-O-(6″-O-acetyl)-β-D-glucopyranoside is a naturally occurring flavonoid found in plants such as Safflower (*Carthamus tinctorius* L. Compositae) seeds, flowers and leaves. It has an m/z of 487 and characteristic fragments of m/z with 473 [M − H-14] ^−^, 451 [M − H-22] ^−^, and 415 [M − H-36] ^−^.

5,7,2′-Trihydroxy-flavanone-4′-O-β-D-glucoside is a naturally occurring flavanone with an m/z of 449 and characteristic fragments of m/z 395 [M − H-54] ^−^, 324 [M − H-71] ^−^, and 323 [M − H-1] ^−^. Likewise, another 5,7,2′-Trihydroxy-flavanone-4′-O-β-D-glucoside with a similar molecular formula but a different chemical structure was detected. It has an m/z of 449 and characteristic fragments of m/z 423 [M − H-26] ^−^, 370 [M − H-53] ^−^, and 267 [M − H-103] ^−^. These compounds are isomers.

Shanciol B is mostly found in *Pleione* (Orchidaceae). It showed a [M − H] ^−^ ion at m/z 421 and MS/MS fragment ions at m/z 385 [M − H-36] ^−^.

Iridin is an isoflavone, a subclass of flavonoid. It is commonly found in several species of irises such as *Iris florentina*, *Iris versicolor*, and *Iris kemaonensis*. It showed a [M − H] ^−^ ion at m/z 521 and MS/MS fragment ions at m/z 485 [M − H-36] ^−^, 467 [M − H-18] ^−^, and 396 [M − H-71] ^−^.

Helonioside B is a phenylpropanoid that derived aromatic amino acids phenylalanine in most plants or tyrosine in partial monocots. It has an m/z of 735 and characteristic fragments of m/z 699 [M − H-36] ^−^, 682 [M − H-17] ^−^, and 357 [M − H-325] ^−^. Similarly, another Helonioside B has been detected with an m/z of 735 and characteristic fragments of m/z 683 [M − H-52] ^−^, 590 [M − H-93] ^−^, and 422 [M − H-168] ^−^.

2,3,5,4′-Tetrahydroxystilbene-2,3-O-β-D-glucopyranoside is an active component of *Polygonum multiflorum* Thunb. (THSG), a Chinese medicinal plant. It showed a [M − H] ^−^ ion at m/z 567 and MS/MS fragment ions at m/z 531 [M − H-36] ^−^, 513 [M − H-18] ^−^, and 437 [M − H-76] ^−^. Similarly, another 2,3,5,4′-Tetrahydroxystilbene-2,3-O-β-D-glucopyranoside has been detected with an m/z of 567 and characteristic fragments of m/z 531 [M − H-36] ^−^, 503 [M − H-28] ^−^, and 439 [M − H-64] ^−^.

Erigoster A is a phenolic compound and it is mostly found in the herbs of *Erigeron speciosus*. It showed a [M − H] ^−^ ion at m/z 557 and there were no MS/MS fragment ions detected for this compound.

Ginsenoside Ra1 is a component of ginseng known as triterpenoids. It showed a [M − H] ^−^ ion at m/z 1209 and MS/MS fragment ions at m/z 1166 [M − H-43] ^−^, 939 [M − H-227] ^−^, and 814 [M − H-125] ^−^.

Asperuloside tetraacetate is an iridoid glycoside that mostly found in *Herba Paederiae*, a component of traditional Chinese herbal medicine. It has an m/z of 581 and characteristic fragments of m/z with 562 [M − H-19] ^−^, 550 [M − H-12] ^−^, and 447 [M − H-103] ^−^.

Nortracheloside is a compound found in lignans. Lignans are polyphenols which mostly found in plants, especially whole grains, seeds and vegetables. It showed a [M − H] ^−^ ion at m/z 535 and MS/MS fragment ions at m/z 529 [M − H-6] ^−^, 395 [M − H-134] ^−^, and 373 [M − H-22] ^−^.

Clinical observations and body weight change.

Tables 2 and 3 shows the body weight changes and the clinical observations of mice in the control group, 10 mL/kg and 2.5 mL/kg kefir treated groups. All mice from the control and kefir treated groups survived until the end of the study without demonstrating any significant changes in the body weight (Table 2) and toxicology symptoms or any abnormal behaviours (Table 3).

**Table 2:** Bodyweight of mice treated with kefir water at high and low dosage

Treatment	Bodyweight (g)				
	Day 0	Day 7	Day 14	Day 21	Day 28
Control	19.35 ± 0.97	21.32 ± 1.12	25.16 ± 1.58	28.88 ± 1.58	29.05 ± 1.53
Kefir 10 mL/kg	20.23 ± 0.64	24.05 ± 0.99	27.38 ± 1.56	29.89 ± 1.49	32.97 ± 1.54
Kefir 2.5 mL/kg	19.26 ± 1.09	23.16 ± 1.03	26.99 ± 0.91	30.88 ± 1.67	31.47 ± 1.5

Values are mean ± SD (*n* = 4) and have been analysed using post hoc comparison test one-way ANOVA. Data revealed no significant difference *p* > 0.05

**Table 3:** Mice health has been monitored and recorded, as shown below

Treatment	Posture	Mobility & Responsiveness	Hair coat	Breathing	Eyes & Nose	Sign of Diarrhea
Control	Normal	Normal	Normal, shiny and smooth	Normal	Clear and open	None
Kefir 10 mL/kg	Normal	Normal	Normal, shiny and smooth	Normal	Clear and open	None
Kefir 2.5 mL/kg	Normal	Normal	Normal, shiny and smooth	Normal	Clear and open	None

### Biochemical analysis

As shown in Table 4, nine serum marker assays were examined to study the toxicological effects of kefir sample in subchronic study. Alanine aminotransferase (ALT), alkaline phosphatase (ALP), aspartate aminotransferase (AST), total protein (TP), albumin (ALB), globulin (Glo) and γ-glutamyl transferase (GGT) were quantified to check on any significant liver injury after kefir water consumption while the creatinine and urea tests were examined for any sign of kidney injury or dysfunction. Based on the results, no significant increase was observed in all serum markers tested when compared to the control group.

**Table 4:** Biochemical profile of BALB/c mice treated with kefir water at high and low dosage

Treatment	ALT (U/L)	ALP (U/L)	AST (U/L)	Creatinine μmol/L	Urea mmol/L	TP g/L	ALB g/L	Glo g/L	GGT U/L
**Control**	19.5 ± 1.5	53.5 ± 1.5	40.5 ± 3.5	18.5 ± 0.5	3.6 ± 0.6	33.6 ± 0.2	17.8 ± 0.8	15.8 ± 0.6	3.0 ± 0.5
**Kefir 10 mL/kg**	18.0 ± 2.0	54.0 ± 0	39.0 ± 3.0	23.0 ± 3.0	3.2 ± 0.1	34.0 ± 0.3	18.5 ± 0.7	16.2 ± 0.3	4.0 ± 0
**Kefir 2.5 mL/kg**	22.0 ± 3.0	53.5 ± 0.5	41.0 ± 1.0	16.0 ± 1.0	2.8 ± 0	36.2 ± 0.4	20.5 ± 0.3	16.3 ± 0.1	< 2 ± 0

### Histopathology of liver, kidney, spleen and brain of BALB/c mice treated with kefir

**Fig. 2:**
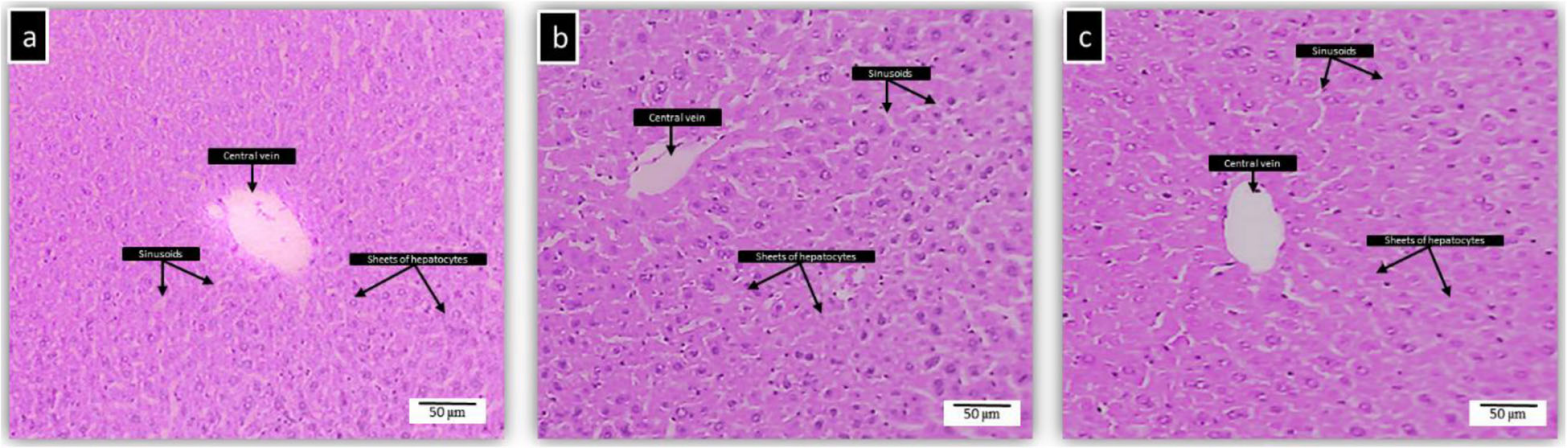
Histopathology of the liver. **a** The liver of male BALB/c mice treated orally with water (control), **b** kefir 10 mL/kg, and **c** kefir 2.5 mL/kg for 28 days. No sign of toxicity was observed in the liver of these mice (× 10 magnification)

**Fig. 3:**
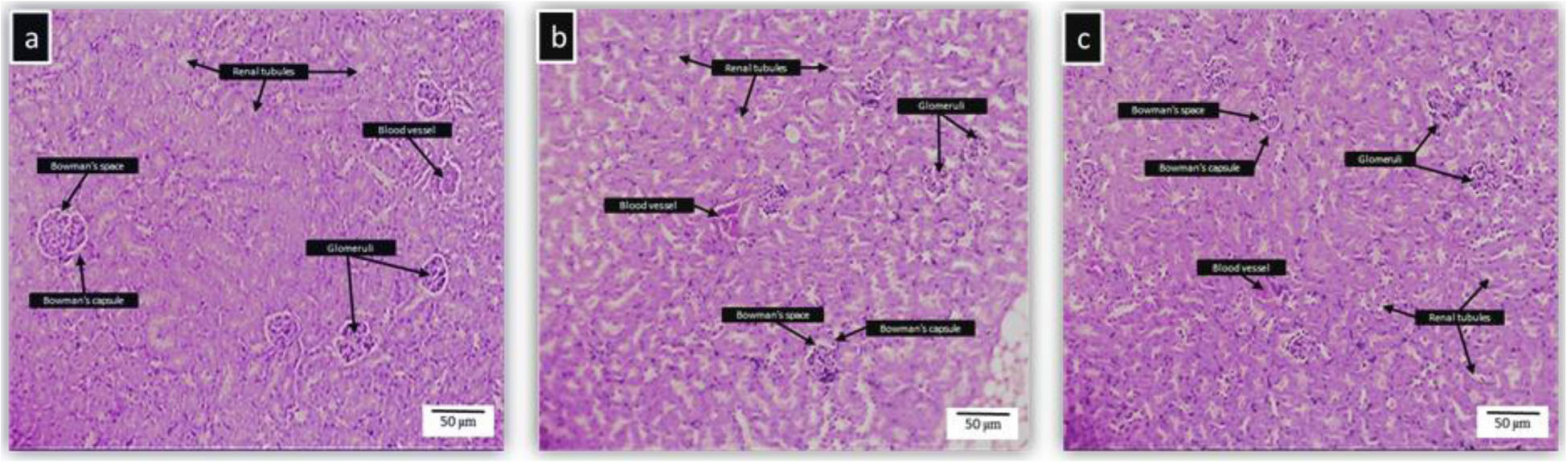
Histopathology of the kidney. **a** The kidney of male BALB/c mice treated orally with water (control), **b** kefir 10 mL/kg, and **c** kefir 2.5 mL/kg for 28 days. No sign of toxicity was observed in the kidney of these mice (× 10 magnification)

**Fig. 4:**
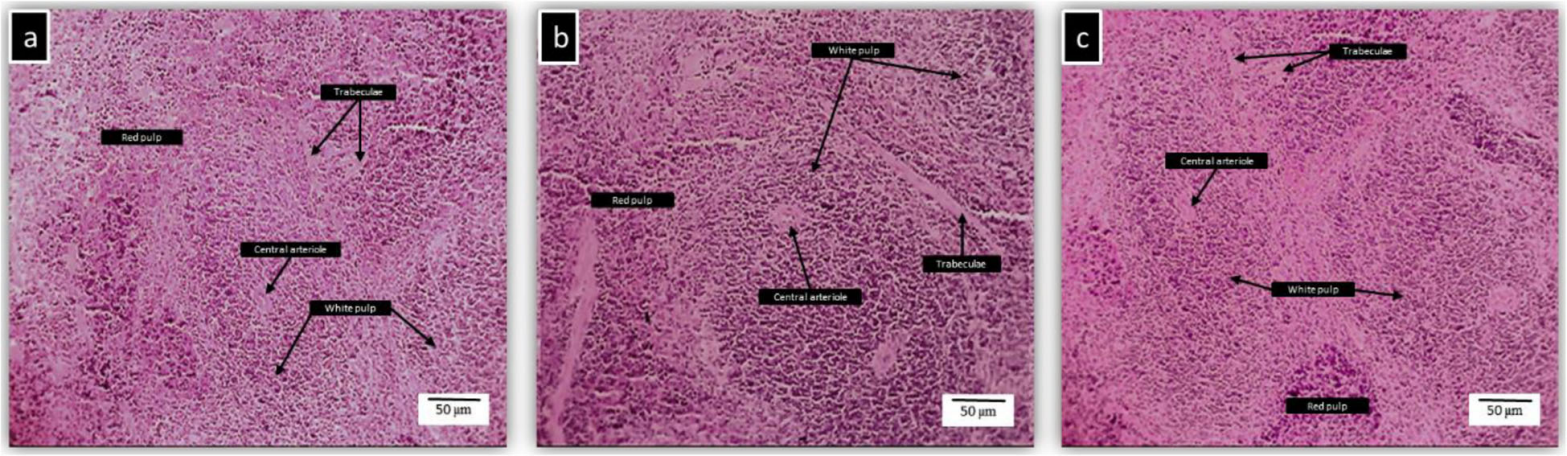
Histopathology of the spleen. **a** The spleen of male BALB/c mice treated orally with water (control), **b** kefir 10 mL/kg, and **c** kefir 2.5 mL/kg for 28 days. No sign of toxicity was observed in the spleen of these mice (× 10 magnification)

**Fig. 5:**
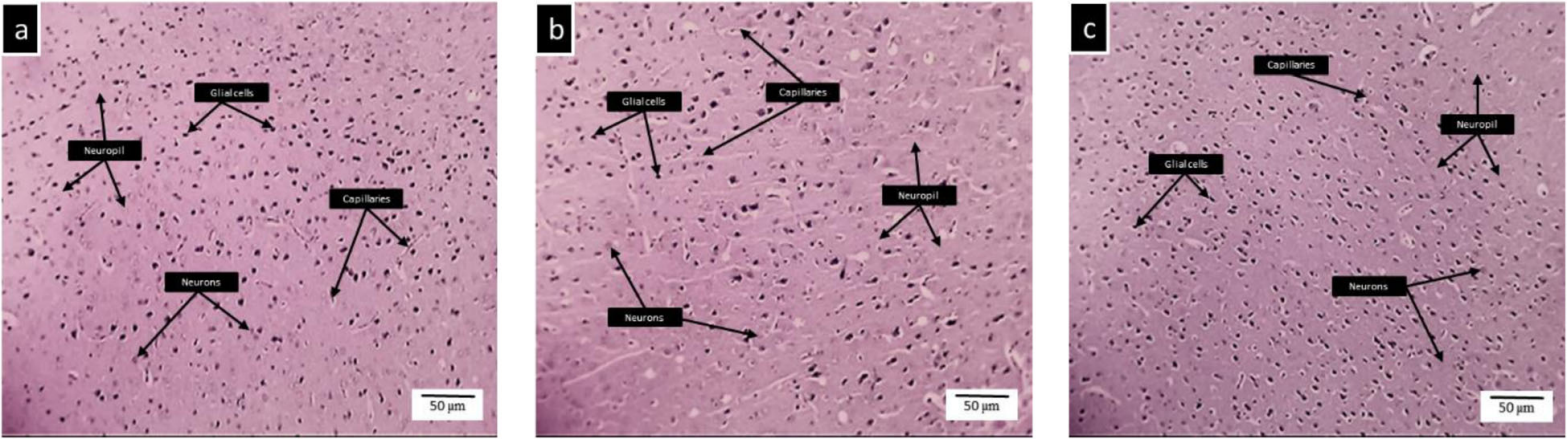
Histopathology of the brain. **a** The brain of male BALB/c mice treated orally with water (control), **b** kefir 10 mL/kg, and **c** kefir 2.5 mL/kg for 28 days. No sign of toxicity was observed in the brain of these mice (× 10 magnification)

Histopathological studies were conducted on four major organs such as liver, kidney, spleen, and brain to confirm the biochemical findings. The photomicrographs of the untreated and kefir treated organs showed normal morphological architecture. Figure 2 shows the histopathology of untreated and kefir treated liver samples. The liver showed normal cellular architecture with binucleation and without any distortions. There were no signs of injury, congestion, necrosis, fatty acid accumulation or haemorrhagic areas around the central vein or sinusoids of the liver. The hepatocytes were clearly visible and still arranged in cords. The liver cross-section showed no lyses in the blood cells, lymphocytes, neutrophil, or macrophage infiltration. Figure 3 shows the histopathology of untreated and kefir treated kidney samples. There were no morphological changes observed in both untreated and kefir treated kidneys of mice. The glomerular architecture appeared normal similar to the control group. The cross-sections of the glomeruli, distal and proximal tubules in the kidney were seemed normal. The cross-section also showed no interstitial and intraglomerular congestion or tubular atrophies. All the nephron cells showed clearly visible and normal nucleoli without bleeding, degeneration or necrosis. Figure 4 shows the histopathology of untreated and kefir treated spleen samples. Based on the cross-section of the spleen, the spleen tissue structure for all groups was normal. There was no sign of pathological change and haemorrhage in the spleen sinus. Figure 5 shows the histopathology of untreated and kefir treated brain samples. The cross-section of the brain samples from both treated and untreated kefir groups showed no histopathological, haemorrhagic, degenerative, congestive, or vacuolar changes. This microscopic evaluation did not reveal any morphological abnormalities that could be attributed to the oral administration of kefir to the mice.

### Antioxidant activity in liver, kidney, spleen and brain samples of BALB/c mice treated with kefir

**Fig. 6:**
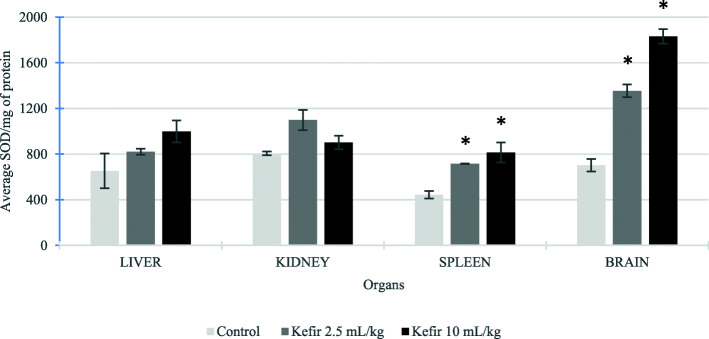
Superoxide dismutase level in liver, kidney, spleen and brain samples from untreated and kefir treated groups. Data are presented as means ± SD. Significant difference from the untreated group was determined using one-way ANOVA and indicated by *p < 0.05

**Fig. 7:**
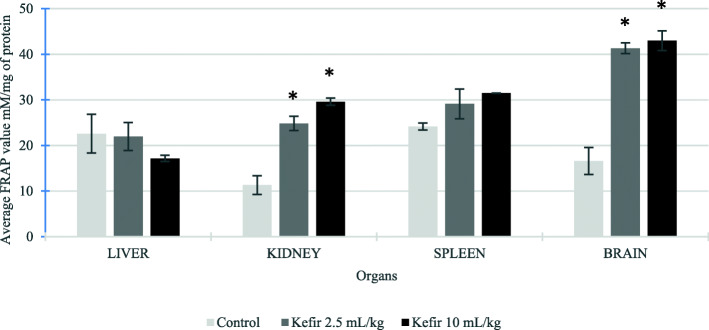
Ferric reducing ability plasma level in liver, kidney, spleen and brain samples from untreated and kefir treated groups. Data are presented as means ± SD. Significant difference from the untreated group was determined using one-way ANOVA and indicated by *p < 0.05

**Fig. 8:**
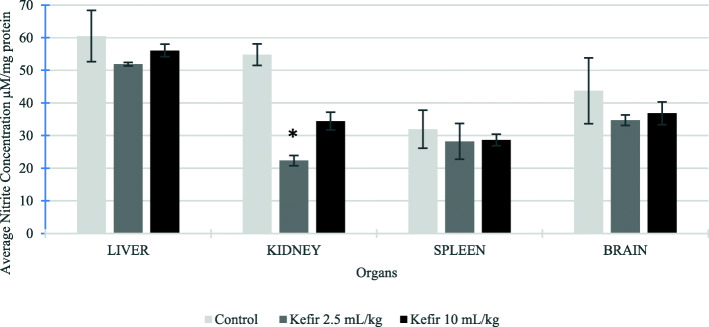
Nitric oxide level in liver, kidney, spleen and brain samples from untreated and kefir treated groups. Data are presented as means ± SD. Significant difference from the untreated group was determined using one-way ANOVA and indicated by *p < 0.05

The antioxidant activities of liver, kidney, spleen and brain homogenates from all treatment groups were examined using SOD and FRAP assays. Based on Fig. 6, the SOD levels were increased in all the organs for both kefir treated groups than the control group. However, a significant increase in SOD level was found in mice brain and spleen samples treated with both kefir doses. The SOD level in brain samples treated with kefir 2.5 mL/kg and 10 mL/kg increased from 703.02 ± 55.09 unit/mg protein to 1353.50 ± 56.32 unit/mg protein and 1830.68 ± 64.43 units/mg protein, respectively. The SOD level in spleen samples treated with kefir 2.5 mL/kg and 10 mL/kg increased from 444.60 ± 32.51 unit/mg protein to 715.65 ± 2.99 unit/mg protein and 814.70 ± 87.10 units/mg protein, respectively. Likewise, the FRAP value (Fig. 7) was significantly increased in brain and kidney samples of the mice treated with both kefir doses: brain FRAP value increased from 16.60 ± 2.97 μM Fe (II)/mg protein to 41.31 ± 1.17 μM Fe (II)/mg protein for kefir 2.5 mL/kg treatment and 42.99 ± 2.15 μM Fe (II)/mg protein for kefir 10 mL/kg treatment; kidney FRAP value increased from 11.31 ± 2.05 μM Fe (II)/mg protein to 24.84 ± 1.53 μM Fe (II)/mg protein for kefir 2.5 mL/kg treatment and 29.59 ± 0.80 μM Fe (II)/mg protein for kefir 10 mL/kg treatment. The result also shows that both the kefir treatments increase the FRAP value slightly in spleen samples compared to the control group. Nevertheless, a slight decrease in the FRAP value was found in liver samples from 22.58 ± 4.24 μM Fe (II)/mg protein to 21.94 ± 3.07 μM Fe (II)/mg protein for kefir 2.5 mL/kg and 17.16 ± 0.68 μM Fe (II)/mg protein for kefir 10 mL/kg, which was not significant. On the other hand, the level of nitric oxide in liver, kidney, spleen and brain lysates were determined using the Griess Reagent Kit. Figure 8 shows an insignificant reduction of NO level in liver, spleen and brain samples treated with both the kefir doses. However, a significant reduction (*p* < 0.05) of NO was observed in kidney samples treated with kefir 2.5 mL/kg, which reduced from 54.82 ± 3.28 μM NO/mg protein to 22.34 ± 1.57 μM NO/mg protein.

## Discussion

The abundance of the *Lactobacillus* genus found in this water kefir grain was consistent with the previous high-throughput sequencing-based kefir studies [19, 36–38]. Likewise, the second most abundant *Acetobacter* genus and the following *Gluconobacter* and *Oenococcus* genera were also detected in several previous kefir studies [19, 38–44]. Though, several studies have reported the genera that resides in kefir grains, studies on *Bacteroides* genera were found very limited. Based on the species level taxonomy tree (Fig. 1), the genus *Lactobacillus* exclusively consisted of *Lactobacillus hilgardii* and *Lactobacillus harbinensis* in which these species were frequently identified in other kefir grains [19, 45–47]. *Lactobacillus hilgardii* and *Lactobacillus harbinensis* are lactic acid bacteria which are hardly found in water kefir. *Lactobacillus hilgardii* is an obligate heterofermentative lactic acid bacteria which mostly found in cocoa and wine fermentations [48, 49]. This bacterial species is known to be the primary producer of exopolysaccharide (EPS) in water kefir ecosystem [47, 50–52]. Nevertheless, not all *Lactobacillus hilgardii* strains produce EPS in water kefir. Other EPS-producing lactic acid bacteria identified from water kefir are included *Leuconostoc mesenteroides, Lactobacillus casei, Lactobacillus brevis, Lactobacillus nagelii*, and *Lactobacillus hordei* [8, 53]. *Lactobacillus harbinensis* is a facultative heterofermentative bacteria which was initially isolated from a Chinese vegetable fermentation [54]. This species was then successively found in the oral ecosystem of healthy individuals [55], sorghum sourdough fermentation [56], Parmigiano Reggiano cheese [57], and French cow milk [58].

Previous studies have demonstrated the ability of *Lactobacillus* species in metabolising flavonoid and phenolic compounds as their end products in fermentation [59–62]. However, this ability is based on species- or strain specific. The enzymatic hydrolysis of lactic acid bacteria fermentation increases both the flavonoid and phenolic compound production; thus, increases the antioxidant activities in the kefir water [63, 64]. In this study, *Lactobacillus* species present in kefir grain are able to produce flavonoid and phenolic compounds which reflects in the UHPLC analysis. The UHPLC screening of kefir water revealed that the major twenty metabolites found were naturally occurring flavonoids and phenolic derivatives (Table 1). These listed flavonoids and phenolic acid derivatives have been studied previously for their high antioxidant, anti-inflammation, and anti-cancer properties [65–75]. For example, the predominant phytochemical detected in kefir water was Trifloroside (isotype match intensity RMS: 1400.16%). It is a β-D-glucoside, a member of phenols, an acetate ester, a delta-lactone, a secoiridoid glycoside, and a pyranopyranone, which mostly found in the roots and herb of Gentiana species. Trifloroside has been reported for its great antioxidant, anti-inflammation, and anti-diabetic potentials. Hexane-ethyl acetate–methanol-water separated 31.15 mg trifloroside with 98.9% purity inhibited nitric oxide production in lipopolysaccharide-induced BV2 cells with high cell viability in a concentration-dependent manner, revealing the usage of the compound as a nitric oxide inhibitor [65]. Likewise, (1S,5R,9R)-deglucosylTrifloroside significantly inhibited lipopolysaccharide-induced nitric oxide production with IC50s of 17.6 μM, showing stronger inhibitory action of indometacin, a clinically used drug [66]. The conversion of Trifloroside into Deglucosyltrifloroside showed a better antioxidative effect in the DPPH assay, Trolox equivalent antioxidant capacity, and reactive oxygen species in HT22 cell assays [67]. Moreover, 50 muM of Deglucosyltrifloroside treatment on 5 mM glutamate-induced HT22 cells showed significant inhibition of the glutamate-induced lactate dehydrogenase leakage, lipid peroxidation, Ca (2+) influx, and the production of intracellular reactive oxygen species, indicating Deglucosyltrifloroside potential in mitigating various oxidative stresses [67]. Besides, Trifloroside has been reported to increase the radical scavenging potential from 20.82–25.14 mg Trolox/g [68]. Furthermore, Gentiana scabra root extract containing Trifloroside showed blood glucose lowering effects on db/db mice through GLP-1 secretion [69]. The finding of the present study is in agreement with Talib et al. (2019), who demonstrated the ability of *Lactobacillus* species isolated from Malaysian kefir grains, including *Lactobacillus harbinensis* in producing high phenolic and flavonoid contents [59]. These flavonoid and phenolic compounds are known to be the main contributors to antioxidant strengths in kefir water. Kefir has been consumed over 100 s of years, and as it is consumed as a traditional drink, no lethality has been reported nor the appropriate dosage for consumption using the kefir water fermented in Malaysia. Thus, it is crucial to evaluate this matter, especially on the dosage and its effects taken over an extended period of time to ensure there are no adverse effects. Based on the subchronic toxicity study, all the kefir treated mice survived until the end of the treatment period without demonstrating any abnormal behaviours or any toxicology symptoms. This finding is in agreement with Diniz et al. (2014) who have stated that normodose and high-dose of kefir supplementation did not cause any significant changes in growth, serum biochemistry, haematology, and histopathology analysis upon oral administration in Wistar rats [76]. Besides, the adverse effects of drugs and chemicals can be detected from the change in body weight [77, 78]. In this study, no significant changes in the body weight were found, indicating no appetite loss symptoms or injury in the oesophagus or stomach that restrained the mice from eating regularly. In fact, the bodyweight of the mice was increased during the 28 days of treatment period, as shown in Table 2, suggesting no toxic effects from the kefir water administration.

Indication of organ injuries can be determined by the elevation of specific biochemical markers in the serum. For example, liver injury can be detected with an increase in the ALT, AST, ALP and total bilirubin level [79, 80], whereas kidney injury or dysfunction can be determined with an increase in creatinine level [81, 82]. Based on the biochemical analysis (Table 4), the markers tested for liver (ALT, ALP, AST, TP, ALB, Glo, and GGT) and kidney (creatinine and urea) functions were in the normal range for both the kefir treatment groups. The insignificance of the serum biochemical results suggests that kefir water samples at both concentrations tested in this study are safe to be consumed. Besides, the histological analyses as shown in Figs. 2, 3, 4 and 5, did not reveal any significant pathological changes in liver, kidney, spleen and brain samples of the mice treated with high and low kefir concentrations. This shows that kefir water at high and low concentrations did not induce any adverse effect on the mice.

In this subchronic study, the regulations of antioxidant activities by kefir water was also evaluated in liver, kidney, spleen and brains samples. Kefir water exhibited a significant increase in the SOD level on brain sample (Fig. 6) and FRAP level on brain and kidney samples (Fig. 7) as compared to the control group. Besides that, there was no significant difference of nitric oxide observed in liver, spleen and brain samples resulting in no inflammation or damage in the evaluated organs (Fig. 8). However, the nitric oxide was significantly reduced in kidney samples treated with 2.5 mL/kg kefir sample. The result showed that 2.5 mL/kg of kefir water is sufficient to reduce the inflammation in the kidney. The results obtained in the present study demonstrated that this kefir water fermented in Malaysia is a good source of antioxidant due to the high levels of flavonoid and phenolic acid derivatives produced by the *Lactobacillus hilgardii* and *Lactobacillus harbinensis* that reside in the kefir grain.

## Conclusion

The 16S rRNA metagenomics evaluation of the kefir grain revealed *Lactobacillus hilgardii* as the predominant and *Lactobacillus harbinensis* as the second dominant species. The UHPLC, on the other hand, showed flavonoid and phenolic acid derivatives as the major groups of compounds found in kefir water and that has been responsible for its antioxidant activities. The safety of this kefir water was proven by the subchronic toxicity study where the study did not show any toxicological signs, behavioural changes, or adverse effects on the experimental mice during the 28 days of treatment period. Kefir water administration in mice resulted in enhanced SOD and FRAP activities, and reduced NO level, especially in the brain and kidney samples. The study indicates that the kefir water is safe for human consumption as a primary source of probiotics and also as a daily health supplement.

## Data Availability

The datasets used and/or analysed during the current study are available from the corresponding author on reasonable request.

## Abbreviations

16 s rRNA: 16 s ribosomal RNA
SOD: Superoxide dismutase
FRAP: Ferric reducing ability plasma
NO: Nitric oxide
NGSm: Next-generation sequencing metagenomics
PCR: Polymerase chain reaction
UHPLC: Ultra-high-performance liquid chromatography
HPLC: High-performance liquid chromatography
GC: Gas chromatography
CE: Capillary electrophoresis
MS: Mass spectrometry
NMR: Nuclear magnetic resonance
TWINS-QTOF-MS: Twins quadrupole time-of-flight mass spectrometry
CTAB: Cetyltrimetthylammonium bromide
OTU: Operational taxonomic unit
